# Medial collateral ligament injuries of the knee in male professional football players: a prospective three-season study of 130 cases from the UEFA Elite Club Injury Study

**DOI:** 10.1007/s00167-019-05491-6

**Published:** 2019-04-04

**Authors:** Matilda Lundblad, Martin Hägglund, Christoffer Thomeé, Eric Hamrin Senorski, Jan Ekstrand, Jón Karlsson, Markus Waldén

**Affiliations:** 1 Institute of Clinical Sciences, Sahlgrenska University Hospital, Sahlgrenska Academy, Gothenburg University, Gothenburg, Sweden; 2 Football Research Group, Linköping University, Linköping, Sweden; 3 Division of Physiotherapy, Department of Medical and Health Sciences, Linköping University, Linköping, Sweden; 4 Department of Health and Rehabilitation, Institute of Neuroscience and Physiology, Sahlgrenska Academy, University of Gothenburg, Gothenburg, Sweden; 5 Division of Community Medicine, Department of Medical and Health Sciences, Linköping University, Linköping, Sweden

**Keywords:** MCL, Soccer, Football, Epidemiology, Bracing, Injection, PRP, MRI

## Abstract

**Purpose:**

Medial collateral ligament (MCL) injury is the single most common traumatic knee injury in football. The purpose of this study was to study the epidemiology and mechanisms of MCL injury in men’s professional football and to evaluate the diagnostic and treatment methods used.

**Methods:**

Fifty-one teams were followed prospectively between one and three full seasons (2013/2014–2015/2016). Individual player exposure and time-loss injuries were recorded by the teams’ medical staffs. Moreover, details on clinical grading, imaging findings and specific treatments were recorded for all injuries with MCL injury of the knee as the main diagnosis. Agreement between magnetic resonance imaging (MRI) and clinical grading (grades I–III) was described by weighted kappa.

**Results:**

One hundred and thirty of 4364 registered injuries (3%) were MCL injuries. Most MCL injuries (98 injuries, 75%) occurred with a contact mechanism, where the two most common playing situations were being tackled (38 injuries, 29%) and tackling (15 injuries, 12%). MRI was used in 88 (68%) of the injuries, while 33 (25%) were diagnosed by clinical examination alone. In the 88 cases in which both MRI and clinical examination were used to evaluate the grading of MCL injury, 80 (92% agreement) were equally evaluated with a weighted kappa of 0.87 (95% CI 0.77–0.96). Using a stabilising knee brace in players who sustained a grade II MCL injury was associated with a longer lay-off period compared with players who did not use a brace (41.5 (SD 13.2) vs. 31.5 (SD 20.3) days, *p* = 0.010).

**Conclusion:**

Three-quarter of the MCL injuries occurred with a contact mechanism. The clinical grading of MCL injuries showed almost perfect agreement with MRI grading, in cases where the MCL injury is the primary diagnosis. Not all grade II MCL injuries were treated with a brace and may thus indicate that routine bracing should not be necessary in milder cases.

**Level of evidence:**

Prospective cohort study, II.

## Introduction

The medial collateral ligament (MCL) acts as the primary biomechanical knee joint valgus restraint at 0° and 30° of knee flexion [1, 2]. Consequently, injury to the MCL is most commonly the result of a valgus impact applied to the lateral knee or a combination of valgus force and external rotation of the tibia [3, 4]. Compared with other knee injuries, such as the anterior cruciate ligament (ACL) injury, the epidemiological literature on MCL injuries in sports is scarce, but they are prevalent in contact sports such as football [5], ice hockey, rugby, wrestling and judo [6–8]. MCL injury has been reported as the most common traumatic knee injury leading to time loss in men’s professional football [9]. A men’s professional football team with a typical 25-player squad will suffer two MCL injuries each season on average, whereas the same team will encounter only one ACL injury every second season [10].

The MCL has greater potential to heal after injury and this, therefore, often leads to full recovery due to its extra-articular location and sufficient vascularisation, in contrast to the ACL [11]. Consequently, isolated grade I and II MCL injuries are treated almost exclusively non-surgically with progressive physiotherapy and sometimes a stabilising knee brace, while grade III injuries may require additional surgical intervention with a ligament repair or reconstruction [1]. The use of a knee brace in the treatment of MCL injury is, however, not supported by high-quality evidence, but it may provide mechanical support and facilitate range of motion training in the early post-injury period, especially in grade II injuries [3, 12–14]. There is a lack of evidence to support the efficacy of platelet-rich plasma (PRP) injections to treat MCL lesions in humans, but there is some emerging evidence to show that the use of PRP injections in the early stages of healing may improve outcome in animal models of acute MCL injuries [5, 15].

The purpose of this prospective cohort study was to describe the epidemiology of MCL injuries in male professional football players, where the MCL injury was reported as the primary injury. Specifically, the primary objective was to study the mechanisms of MCL injury, while the secondary objectives were to evaluate the diagnostic and treatment methods used by the medical teams, such as imaging, bracing and injection therapy and to study the agreement between clinical evaluation and MRI when grading the severity of the injury.

## Materials and methods

This is a sub-study in a long-term prospective cohort study carried out in collaboration with the Union of European Football Associations (UEFA), the so-called UEFA Elite Club Injury Study, investigating men’s professional football in Europe since 2001 [16]. For the purpose of this study, 51 teams with 2018 individual players from the highest national leagues in 17 European countries were followed for between one and three seasons between July 2013 and May 2016. All contracted players listed in the first team squads each season were invited to participate in the study. Players who left the team during the season were only included while playing with the team. The full methodology and the development of the study design has previously been reported in detail [17]. The overall study design followed the consensus on definitions and data collection procedures in studies of football injuries [18].

### Data collection

Baseline data regarding anthropometrics and dominant leg (preferred kicking leg) were collected at player inclusion each season. Individual player exposure during training and matches was registered in minutes by the teams’ medical staff on a standard exposure form sent to the study group every month. The teams’ medical staff also recorded time-loss injuries on a standard injury form that was sent to the study group each month. This general injury form provided information about the diagnosis, nature and circumstances of injury occurrence. When an MCL injury was reported as the main diagnosis on the general injury form, an additional study-specific MCL injury form was e-mailed to the team’s medical staff requesting details on previous knee ligament injuries, clinical grading, imaging findings and specific treatment details [19]. Consequently, there were no MCL injuries with, for example concomitant cruciate ligament injury included in this sub-study (such injuries were classified with the cruciate ligament injury as the main diagnosis). The player was regarded as injured until the team medical staff allowed full participation in training and availability for match selection. All injuries were followed until the final day of rehabilitation.

### Definitions and grading

An overview of the general definitions used in the study is given in Table 1. MCL injury was defined as ‘a traumatic distraction injury to the superficial MCL (sMCL), deep MCL (dMCL) or the posterior oblique ligament (POL) leading to a player being unable to participate fully in training or match play’. The MCL injuries were categorised into three different severity grades based on findings during clinical examination, magnetic resonance imaging (MRI) or other diagnostic utilities. Only clinical and MRI gradings were used for analyses in this study. The clinical grading system on the MCL injury form was based on the medial joint opening during the valgus stress test in semi-flexion and full extension, i.e. medial knee joint laxity [20]. On MRI, a grade I injury is characterised by intact fibres with surrounding oedema, a grade II injury as partial fibre disruption and a grade III injury as complete fibre disruption or avulsion [21]. There was no predefined order in which the MRI and clinical grading of MCL injuries were performed. Whether MRI was performed or not following injury was a decision completely in the hands of the club medical teams without any pre-defined criteria or specific algorithm in the study manual.

**Table 1:** Operational definitions used in the study

Training session	Team training that involved physical activity under the supervision of the coaching staff
Match	Competitive or friendly match against another team
Injury	Injury resulting from playing football and leading to a player being unable to participate fully in future training or match play (i.e. time-loss injury)
Rehabilitation	A player was injured until team medical staff allowed full participation in training and availability for match selection
Re-injury	Injury of the same type and at the same site as an index injury occurring no more than two months after a player’s return to full participation from the index injury
MCL injury grading	
Clinical grade I	Tenderness on palpation or pain during stress test but no or only minimally increased laxity
Clinical grade II	Increased laxity during stress test with semiflexion but not in full extension
Clinical grade III	Gross laxity during stress test with semiflexion and increased laxity also in full extension
MRI grade I	Oedema/haemorrhage within or surrounding the ligament but intact fibres
MRI grade II	Partial ligamentous disruption but with continuity and some intact fibres
MRI grade III	Complete ligamentous disruption or osseous avulsion, discontinuity and virtually no intact fibres
Traumatic injury	Injury with sudden onset and known cause
Overuse injury	Injury with insidious onset and no known trauma
Non-contact injury	Injury occurring without any contact with another player or object
Contact injury	Injury occurring with contact with another player or object
Injury rate	Number of injuries per 1000 player hours [(Σ injuries/Σ exposure hours) × 1000]

### Ethical approval

The study design was approved by the UEFA Medical Committee and the UEFA Football Development Division. All players provided written informed consent prior to participation.

### Statistical analysis

All analyses were performed using SAS software version 9.4 (SAS Institute Inc, Cary, NC, USA) and SPSS (IBM SPSS Statistics for Windows, Version 24.0. Armonk, NY, USA: IBM Corp.). Lay-off time was reported in days as the mean and standard deviation (SD). The use of injections was only reported stratified for grade II MCL injuries determined from MRI, as the number of patients with grade I and III injuries who received injection therapy was low. For between-group comparisons, the Mann–Whitney *U* test was used for continuous variables, Fisher’s exact test was used for dichotomous variables and the Chi-squared test was used for non-ordered categorical variables. Agreement between MRI and clinical grading was described by percentage agreement and weighted kappa with 95% CI. The following kappa cut-off values were used; ≤ 0.20 corresponds to slight agreement, 0.21–0.40 fair, 0.41–0.60 moderate, 0.61–0.80 substantial and 0.81–1 almost perfect agreement [22]. The systematic differences between the methods were analysed with the sign test. All tests were two-tailed and conducted at the 0.05 significance level.

## Results

During the three seasons that were studied, a total of 4364 injuries were registered, of which 130 injuries (3.0%), in 115 players, had MCL injury as the main diagnosis on the general injury form. Eleven of the 130 MCL injuries (8.5%) were re-injuries with a recurrent ipsilateral MCL injury within 2 months of return to play (RTP). Four of the players suffered an MCL injury in both knees on separate injury occasions during the observation period.

### Characteristics and mechanisms of injury

A total of 98 (75.4%) MCL injuries had a contact injury mechanism, where being tackled (*n* = 38, 29.2%) and tackling (*n* = 15, 11.5%) were the two most common playing situations (Table 2). Of the 115 players who sustained an MCL injury, four (3.5%) reported a previous MCL injury to the ipsilateral knee on the specific MCL injury form. The vast majority of the MCL injuries were reported as isolated lesions (*n* = 114, 87.7%). Sixteen of the MCL injuries (12.3%) had associated lesions to the ipsilateral knee, where damage to the medial meniscus was most frequent (*n* = 4, 3.1%), followed by cartilage lesions (*n* = 3, 2.4%). In addition, there was no significant difference in the MRI grading of the contact vs. non-contact injuries (n.s.).

**Table 2:** Contact injury mechanisms

	Training			Match			Total (%)
	Contact object	Contact player	Non-contact	Contact object	Contact player	Non-contact	
Being tackled	0	7	0	0	31	0	38 (29.2)
Tackling	0	3	0	0	12	0	15 (11.5)
Collision	0	5	0	0	7	0	12 (9.2)
Twisting/turning	0	0	6	0	1	2	9 (6.9)
Blocked	0	4	0	0	5	0	9 (6.9)
Kicked	0	4	0	0	3	0	7 (5.4)
Shooting	0	0	2	1	3	1	7 (5.4)
Other	6^a^	0	13	0	6	8	33 (25.4)
Total	6	23	21	1	68	11	130 (100)

^a^Contact with object such as ball, goalpost or billboard, etc

### Injury lay-off times, grading and location

The mean lay-off period for all 130 MCL injuries was 24 (SD 22) days. The most common clinical grade was grade I and the mean lay-off period for this severity was 10 (SD 32) days.

The most common MCL injury location was the upper third of the ligament (*n* = 68, 54.0%), with a mean lay-off of 23 (SD 21) days. Injuries to the middle third were reported in almost one-third of cases (*n* = 39, 31.0%), with a mean lay-off of 24 (SD 20) days. Injuries to the lower third were less common (*n* = 19, 15.1%) and had a mean lay-off of 24 (SD 29) days. There were no differences in terms of MCL injury locations and lay-off periods (n.s.).

### Clinical and MRI grading

An MRI was used to establish the diagnosis in 71 (54.6%) of the MCL injuries and in conjunction with ultrasonography (US) in another 15 (11.5%) injuries, while US was used solely in four (3.1%), all of which were clinical grade I MCL injuries (Table 3). The MCL injury diagnosis was made by clinical examination exclusively without additional imaging in 33 (25.4%) injuries, all but one being clinical grade I (Table 3). For the 88 players in whom grading was determined by both MRI and clinical examination (Table 4), 80 (92.0% agreement) injuries were equally evaluated, giving a weighted kappa of 0.87 (95% CI 0.77–0.96). Five (5.7%) players had clinical grading that was less severe and two (2.3%) had more severe clinical grading than the MRI grading.

**Table 3:** Diagnostic evaluation methods and clinical grading

	Clinical grading			
	I	II	III	Total
Clinical examination only	32	1	0	33
MRI	29	38	4	71
US	4	0	0	4
MRI and US	7	8	0	15
Radiograph	1	0	0	1
MRI and radiograph	1	0	0	1
Arthroscopy, MRI, US	0	0	1	1
Total	74	47	5	126

Data missing from three injuries on diagnostic evaluation and one on clinical grading

*MRI* magnetic resonance imaging, *US* ultrasonography

**Table 4:** Agreement between clinical and MRI grading of MCL injuries

Clinical grading	MRI grading			
	I	II	III	Total
I	33	4	0	37
II	2	43	1	46
III	0	0	5	5
Total	35	47	6	88

*MRI* magnetic resonance imaging

### Bracing and injection treatment

A total of five of the 75 (6.7%) clinical grade I, 25 of the 47 (53.1%) clinical grade II and all five clinical grade III MCL injuries were treated with a stabilising knee brace. The lay-off period was significantly longer for grade II injuries with a stabilising knee brace compared with grade II injuries without bracing [42 (SD 13) days vs. 32 (SD 20 days, *p* = 0.01)]. No similar analysis was made of grade I injuries, because of few injuries in the bracing group, or of grade III injuries, because they were all treated with bracing.

Thirty-two (25.0%) MCL injuries were treated with injection therapy, most frequently PRP injections, followed by corticosteroids in five patients (3.8%). Two players with MRI grade I, 17 players with MRI grade II and one player with MRI grade III MCL injuries were treated with PRP injections. There were no differences in lay-off times in players treated with PRP or not, in grade II MCL injury grading (n.s.). The other injection therapies were not analysed due to the small number of uses.

### MCL repair

Two of the 130 MCL injuries (1.5%) were treated surgically. The first was a non-contact clinical and MRI grade III injury with a bony avulsion in the upper third of the ligament. This player had had a previous injury to the ipsilateral knee (ACL reconstruction and MCL repair). A stabilising knee brace was used for 6 weeks after the surgical repair. The player returned to play 85 days after surgery. The other MCL rupture leading to surgery was a contact clinical and MRI grade III injury to the lower third of the MCL ligament, with associated cartilage damage. This player had no previous ipsilateral knee injury. A stabilising knee brace was used for 5 weeks after the surgical repair. The player returned to play 119 days after the surgery.

## Discussion

The most important finding in this study was that more than three in four injuries were contact injuries, in which being tackled and tackling were the most common situations that led to injury. Moreover, MRI and the clinical grading of grade I, II and III MCL injuries showed almost perfect agreement. There was an increased lay-off period in players who had sustained a grade II MCL injury treated with a stabilising knee brace compared with the players who did not use a stabilising knee brace. However, it is not known whether this association is related to the treatment with a brace per se, or whether the relationship is related to between-team differences in treatment.

There was a 3:1 relationship between contact (75.4%) and non-contact (24.6%) injury mechanisms in the current study. The contact injuries were primarily sustained in playing situations in which the football player was tackled (29.2%) or tackling (11.5%). Moreover, the vast majority (88%) of the MCL injuries were classified as isolated injuries without concomitant meniscal or cartilage lesions, which confirms that a direct trauma to the knee joint appears to be characteristic of MCL injuries. In contrast, non-contact loaded rotational trauma of the knee joint may instead result in more severe injuries, commonly involving intra-articular structures such as the ACL and the menisci. In fact, only two MCL injuries in this study were sustained during non-contact twisting/turning, which is a well-known injury mechanism for ACL injuries and combined knee ligament injuries [2, 23].

MRI was used to establish the diagnosis in more than three of five of the cases, while one in four were diagnosed by clinical examination only. The main reasons for using imaging techniques were primarily to estimate the prognosis or to determine whether there were any concomitant meniscal or cartilage injuries and not necessarily to verify the clinical diagnosis of the MCL injury. However, in recent years, the prognostic use of MRI has been questioned, especially as a tool for determining return to play. For instance, in hamstring injuries, the use of MRI has not been a useful tool for the clinician in determining when an athlete will be able to return to sport [24], or reducing the risk of re-injury [25]. Whether this applies to collateral ligament injuries remains unknown. However, there was almost perfect agreement between MRI grading and the clinical grading of MCL injuries in the elite football clubs included in this study. The agreement between MRI and clinical grading offers the potential to reduce team expenses for radiographic imaging. The present study also demonstrated a significant relationship between the severity of MCL injury and days to RTP, which is in agreement with previous literature [9]. This indicates a high level of knowledge with regard to the clinical grading of MCL injuries by the medical personnel at the top-level elite clubs. The high-quality clinical examinations by medical teams at elite football clubs suggest a good understanding of the anatomy and biomechanics of the medial structures of the knee, which aids in determining the full extent of injury and may guide the treatment of the respective injury patterns. However, there are still some concerns about missing or underestimated clinical and radiographic findings on the medial side of the knee [26–28].

Players who sustained an MCL grade II injury and were treated with a stabilising knee brace had a lay-off time that was more than 2 weeks longer than that of players treated without a knee brace. However, there are different levels of severity among the grade II injuries (ranging from grade “II− to II+”), which may influence the clinicians’ decision on when to recommend the use of a stabilising knee brace, e.g. when injuries were considered more severe, the players were perhaps braced to a greater extent. The factors that might have influenced the medical teams’ decision about using, or not using, a knee brace are not known. One limitation with our analysis is that different clubs may have different traditions when it comes to recommending or not recommending a knee brace. It is prohibited to play with a knee brace in professional football, unlike ice hockey and American football, where it is permitted to return to play with a hinge brace. The use or lack of a hinged knee brace may affect the lay-off period and comparisons between the lay-off between different sports may therefore be misleading.

The football players in this cohort returned to play within a relatively short period, which suggests that the rehabilitation treatment was good and in line with what previous studies have reported after isolated MCL injuries [23, 27]. The MCL is an important stabiliser of the knee and, when injured in combination with the ACL, surgical treatment of the MCL may be necessary to restore knee joint stability [29], as a small increase in medial laxity may entail an increased risk of secondary consequences, such as the risk of ACL re-rupture and revision [30].

A quarter of the MCL injuries underwent injection therapy as a part of the treatment; over 60% of these injections were PRP. There was no difference in lay-off time in the players treated with or without PRP injections, independent of MCL injury grading. It is not known why the physicians in the elite football teams were prone to use PRP injections or what indicated this treatment, considering the absence of evidence supporting the treatment method in ligament injuries and acute muscle injuries [31]. One of the factors that may influence the clinicians’ decision about whether or not to use PRP injections is that clubs have different traditions when it comes to the treatment strategies they use.

The strength of this study was the large volume of data on MCL injuries collected prospectively from a homogeneous group of male professional football players. This study also has some limitations in addition to those already discussed. First, it only includes MCL injuries that are recorded as the primary diagnosis, thereby limiting this study’s ability to draw conclusions with regard to the impact of sustaining a concomitant MCL injury with, for example, a cruciate ligament injury. Second, the order in which the clinical and MRI grading of MCL injuries was performed may have varied between teams and clinicians and this may have affected the reported grading of the injuries. It would be desirable if all clinical grading of the severity of MCL injuries was reported before MRI grading was determined. Third, the limited variation regarding different treatments and types of examination could restrict the interpretation of data and weaken the statistical tests on the different grades of MCL injuries, which may have been underpowered. Fourth, analyses of MCL re-injuries were not performed, as these analyses would have been underpowered. Finally, possible differences in the choice of treatment options for MCL injuries, including how to determine the diagnosis, the use of MRI, bracing, using injection therapy, rehabilitation and criteria for RTP, could have influenced the interpretation of some of the results.

## Conclusion

Most MCL injuries occurred with a contact mechanism, where the two most common playing situations were being tackled and tackling. The clinical grading of MCL injuries in elite football showed almost perfect agreement with MRI grading, in cases where the MCL injury is the primary diagnosis.
